# Adult Scoliosis Following Intrathecal Baclofen Therapy

**DOI:** 10.7759/cureus.20937

**Published:** 2022-01-04

**Authors:** Hiroshi Fujioka, Hideki Harada, Eiichirou Urasaki

**Affiliations:** 1 Department of Neurosurgery, Cognitive and Molecular Research Institute of Brain Diseases, Kurume University, Fukuoka, JPN; 2 Department of Neurosurgery, Himeno Hospital, Fukuoka, JPN; 3 Neuroanesthesia Research Laboratory, Cognitive and Molecular Research Institute of Brain Diseases, Kurume University, Fukuoka, JPN; 4 Department of Neurosurgery, Fukuoka Mirai Hospital, Fukuoka, JPN

**Keywords:** iatrogenic, spasticity, adult, scoliosis, intrathecal baclofen

## Abstract

In this case report, we present an adult case of scoliosis following intrathecal baclofen (ITB) therapy. A 56-year-old female with stroke-induced right spastic hemiparesis for seven years underwent implantation of an ITB pump. Satisfactory spasticity control was achieved using 30 µg/day of baclofen; however, she began to complain of lumbar pain in the postoperative year (POY) 1. Scoliosis, which was not recognized preoperatively, was confirmed in POY 2 (Cobb angle of 19 degrees). It further progressed into a walking disturbance in POY 5 (Cobb angle of 28 degrees). Hence, posterior fusion with decompression was planned. Following the removal of the ITB pump, spasticity management was replaced by Botox injection. However, the progression of scoliosis and neurological conditions stabilized after the removal, and decompression surgery was electively performed in POY 6. Scoliosis remained stable during the two-year follow-up period (Cobb angle of 28 degrees). This case demonstrates the potential risk of ITB-induced scoliosis in an adult patient. Careful preoperative investigations and postoperative follow-up are recommended for patients on ITB therapy.

## Introduction

Intrathecal baclofen (ITB) therapy is an effective, established neuromodulatory therapy for severe spasticity of central or spinal origin; however, it has the risk of complications associated with the hardware, surgery, and drug itself [1]. There is an ongoing debate regarding the potential risk of scoliosis in children [2-4]. The risk of scoliosis has been reported by case series or case-controlled studies [5,6]; however, contrary findings have also been reported [7,8]. All possible cases of ITB-induced scoliosis have been reported in children, with little information available for adults. Here, we describe a case of ITB-induced scoliosis in an adult patient along with long-term follow-up.

## Case presentation

**Figure 1:**
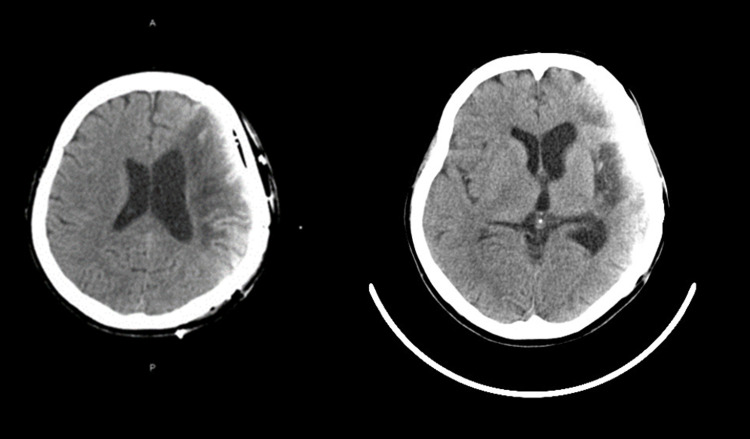
Preoperative head CT. Plain head CT showed old vascular lesions over the left hemisphere, which were associated with right spastic paresis. CT: computed tomography

**Figure 2:**
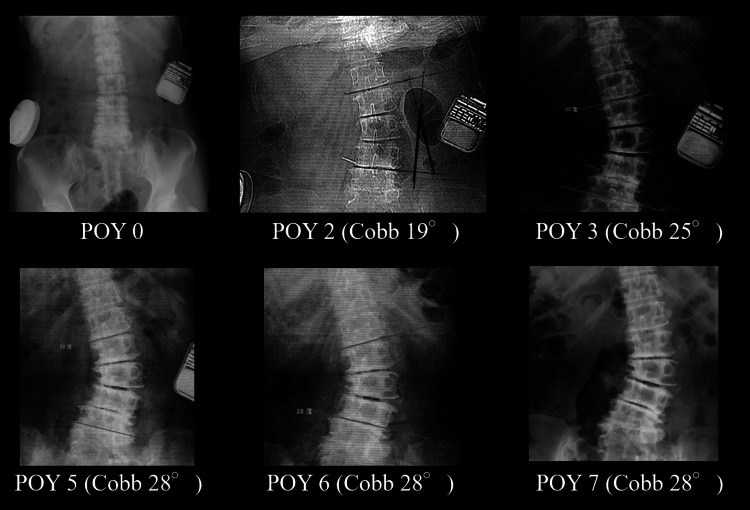
X-rays before and after ITB therapy. Compared to the X-ray performed in POY zero, scoliosis was evident in POY two. Following the removal of the ITB pump in POY five, decompression surgery was electively performed in POY six. Thereafter, the progression of scoliosis ceased for two years and Cobb angle remained at 28 degrees. The rectangular object in the left abdomen is an implantable pulse generator for motor cortex stimulation. ITB: intrathecal baclofen; POY: postoperative year

A 56-year-old female with a history of subarachnoid hemorrhage seven years ago (Figure 1) was referred to our hospital for the management of spasticity over the right limbs. She had right spastic hemiparesis, and spasticity was 1.5-2 points in the upper and 1.5 points in the lower limbs according to the modified Ashworth scale (MAS). She could perform her activities of daily life and was able to walk outside with a single cane. Her body mass index was 23.8 kg/m² (height 164.0 cm, weight 64.0 kg). She had undergone an epidural motor cortex stimulation surgery for right upper limb pain one year previously, which resulted in moderate pain control. Preoperative lumbar X-rays (Figure 2) and bone mineral density tests were unremarkable.

Following an intrathecal screening test, she underwent implantation of an ITB pump (Figure 2, postoperative year [POY] zero) with the intrathecal catheter tip placed at the upper thoracic level. Because baclofen concentration of over 35.0 µg/day caused a mild feeling of weakness on the affected side, 30.0 µg/day was continuously administered thereafter. ITB therapy ameliorated the patient’s spasticity (MAS 1-1.5 points), spasticity-associated pain, and upper limb movements to a modest degree.

Although she was satisfied with ITB therapy, she began to complain about low back pain in POY one. The pain was initially tolerable, and observation therapy was adopted. However, it gradually worsened, and a lumbar X-ray in POY two indicated scoliosis with a Cobb angle of 19 degrees (Figure 2). Gradually, scoliosis progressed into left radicular pain (L4, S1) and walking disturbance, and surgical intervention was indicated in POY five. Our initial surgical plan was a combination of multi-vertebral decompression and posterior fusion. Therefore, following the removal of motor cortex stimulation devices and the ITB pump, spasticity control was achieved by Botox injection.

**Figure 3:**
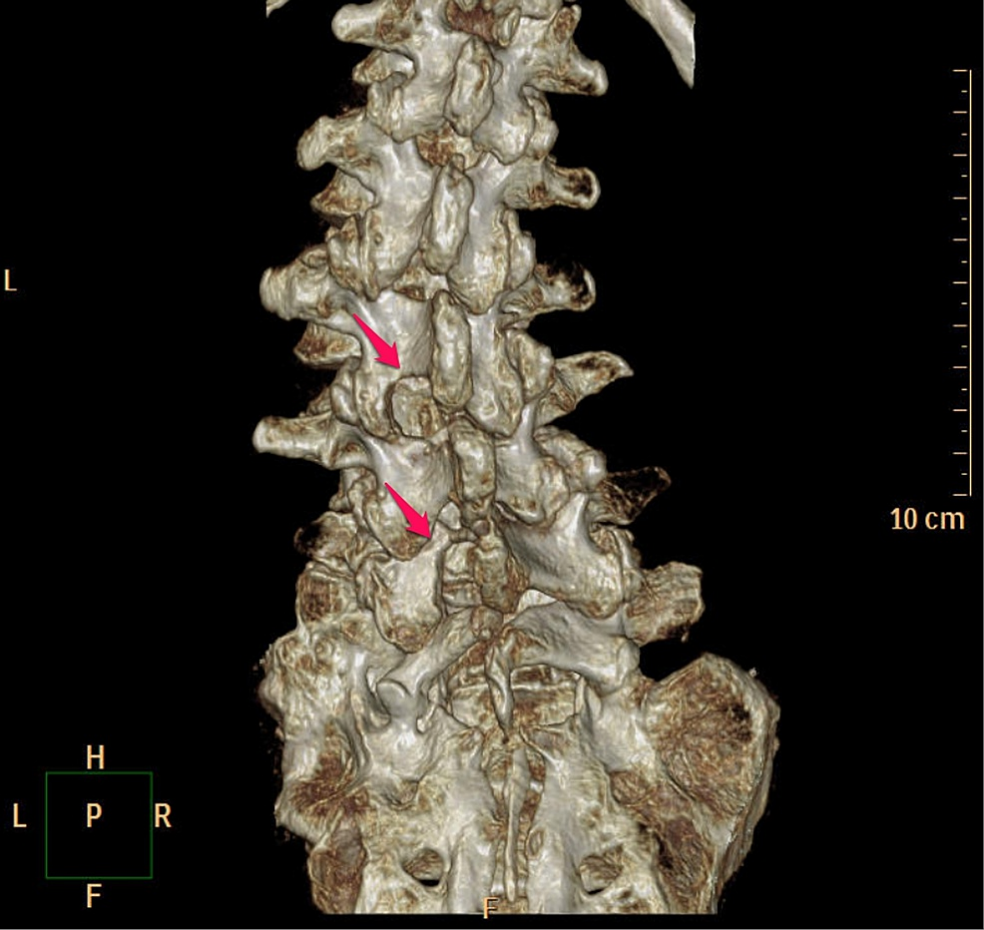
3D-CT after decompression surgery. Decompression was performed at the left L3/4 and L5/6 vertebral levels. 3D-CT: three-dimensional computed tomography

After the removal of the devices, both neurological deterioration and the progression of scoliosis stabilized, following which surgery was rescheduled. In POY six, endoscopic decompression surgery was electively performed at the left L3/4 and L5/6 vertebral levels (Figure 3). A favorable postoperative course was noted, and she was able to walk with a single cane (Cobb angle 28 degrees). Thereafter, no apparent progression of scoliosis was observed for two years on lumbar X-ray and computed tomography.

## Discussion

The potential risk of scoliosis in patients undergoing ITB therapy is controversial [9,10]. As summarized in Table 1, the risk has been described in case series and case-controlled studies [11]. Negative results were reported by a case-matched study [12] and a prospective cohort study [8]. A cohort study by Shilt et al. demonstrated no significant difference in the rate of Cobb angles between an ITB group and a control group [8]; however, the method used to identify the control group may have been biased (see [9] for discussion). To make the issue more challenging, the majority of children who receive ITB therapy have spasticity due to cerebral palsy, and cerebral palsy in children is commonly associated with scoliosis [13] (6.5-76% [9]). Thus, further multicenter, prospective studies are needed.

**Table 1:** Affirmative and negative results for the potential risk of ITB-induced scoliosis. ITB: intrathecal baclofen

Article	Reference	Study design	Results
Segal et al.	[10]	Case series	Undetermined
Sansone et al.	[2]	Case series	Affirmative
Senaran et al.	[7]	Retrospective case-matched review	Negative
Ginsburg et al.	[5]	Case series	Affirmative
Motta et al.	[11]	Retrospective case review	Affirmative
Shilt et al.	[8]	Prospective case-matched study	Negative
Burn et al.	[6]	Retrospective case review	Affirmative
Rushton et al.	[12]	Retrospective matched cohort study	Negative
Walker et al.	[3]	Retrospective, case-matched review	Affirmative
Lins et al.	[4]	Retrospective comparative study	Affirmative
Oh et al.	[14]	Single case report	Affirmative

Little published data are available for the potential risk of ITB-induced scoliosis in adults. One case report in an adult patient demonstrated ITB-induced scoliosis [14]; however, care must be taken while interpreting the results of the study because the patient had stiff-person syndrome, an extremely rare neurological disorder. One of the possible reasons for the paucity of relevant data is its low incidence. Furthermore, because scoliosis in the adult population is common, with a prevalence of 6% above 50 years of age [15], adult scoliosis in patients undergoing ITB therapy has the potential risk to be overlooked if it is asymptomatic. Hence, further research is necessary.

Adult scoliosis, defined as a spinal deformity in a skeletally mature patient with a coronal Cobb angle of more than 10 degrees [15], can be neuromuscular scoliosis, degenerative idiopathic scoliosis, or de novo scoliosis [16]. Although our patient had a history of subarachnoid hemorrhage, neuromuscular scoliosis was considered unlikely as there were no signs of scoliosis for seven years. Degenerative idiopathic scoliosis was also unlikely as the patient did not have a history of scoliosis. Although the possibility of de novo scoliosis cannot be completely negated, there was a long-term causal relationship of scoliosis after ITB therapy and the removal of an ITB pump ceased the progression of scoliosis. Thus, we assumed the involvement of ITB therapy, which suggests iatrogenic scoliosis.

Among likely ITB-induced scoliosis in children, the potential involvement of skeletal immaturity has been implicated [3]. ITB therapy, when applied chronically, may be associated with muscle weakness [17]. As the potential involvement of the paravertebral muscles has also been implicated in adult scoliosis [18], it is likely that ITB therapy contributes to scoliosis through a weakness in the muscle, especially paravertebral muscles.

Because the risk of ITB-induced scoliosis is yet to be established, this case raises an important issue of how to deal with the progression of scoliosis in patients undergoing ITB therapy. Although we suspected the potential involvement of ITB therapy in the progression of scoliosis, we removed the pump primarily because of the assumption that it may interfere with fusion and decompression surgery. Observation is a practical option but is associated with a risk of therapeutic delay. Another option is tentatively tapering the concentration of baclofen, or the temporal cessation of baclofen and, if available, replacement by Botox injection. Careful therapeutic planning is required when scoliosis is identified in patients undergoing ITB therapy.

## Conclusions

The risk of scoliosis in patients undergoing ITB therapy has been controversial. While the potential risk has been suggested in children, little information is available in adults. This case demonstrated the long-term causal relationship of scoliosis after ITB therapy and removal of an ITB pump ceasing the progression of scoliosis. Although further investigation is required, the findings of this study add to the possibility of ITB-induced iatrogenic scoliosis in adult patients.

One of the important issues in patients undergoing ITB therapy is how to deal with the progression of scoliosis. Conceivable therapeutic planning includes observation, tapering the concentration of baclofen concentration, or replacement by Botox injection. With this possibility in mind, preoperative spinal investigation and postoperative follow-up through periodical imaging and neurological examination are desirable for patients undergoing ITB therapy. Further clinical and experimental studies are needed.
